# Development and validation of a glass-silicon microdroplet-based system to measure sulfite concentrations in beverages

**DOI:** 10.1007/s00216-018-1516-6

**Published:** 2019-01-14

**Authors:** Yannick Vervoort, Rodrigo Sergio Wiederkehr, Michiel Smets, Maarten Fauvart, Tim Stakenborg, Gabrielle Woronoff, Liesbet Lagae, Kevin J. Verstrepen

**Affiliations:** 1 Laboratory for Systems Biology, VIB Center for Microbiology, Gaston Geenslaan 1, 3001 Leuven, Belgium; 2 KU Leuven Department M2S, Laboratory for Genetics and Genomics, Gaston Geenslaan 1, 3001 Leuven, Belgium; 3 Imec Life Sciences and Imaging, Kapeldreef 75, 3001 Leuven, Belgium; 4 KU Leuven Department of Physics and Astronomy, Celestijnenlaan 200 D, B-3001 Leuven, Belgium

**Keywords:** Droplet microfluidics, Sulfite, *Saccharomyces pastorianus*, *Saccharomyces cerevisiae*, Fluorescence, Microdroplets

## Abstract

Sulfite is often added to beverages as an antioxidant and antimicrobial agent. In fermented beverages, sulfite is also naturally produced by yeast cells. However, sulfite causes adverse health effects in asthmatic patients and accurate measurement of the sulfite concentration is therefore very important. Current sulfite analysis methods are time- and reagent-consuming and often require costly equipment. Here, we present a system allowing sensitive, ultralow-volume sulfite measurements based on a reusable glass-silicon microdroplet platform on which microdroplet generation, addition of enzymes through chemical-induced emulsion destabilization and pillar-induced droplet merging, emulsion restabilization, droplet incubation, and fluorescence measurements are integrated. In a first step, we developed and verified a fluorescence-based enzymatic assay for sulfite by measuring its analytical performance (LOD, LOQ, the dynamic working range, and the influence of salts, colorant, and sugars) and comparing fluorescent microplate readouts of fermentation samples with standard colorimetric measurements using the 5,5′-dithiobis-(2-nitrobenzoic acid) assay of the standard Gallery Plus Beermaster analysis platform. Next, samples were analyzed on the microdroplet platform, which also showed good correlation with the standard colorimetric analysis. Although the presented platform does not allow stable reinjection of droplets due to the presence of a tight array of micropillars at the fluidics entrances to prevent channel clogging by dust, removing the pillars, and integrating miniaturized pumps and optics in a future design would allow to use this platform for high-throughput, automated, and portable screening of microbes, plant, or mammalian cells.

**Electronic supplementary material:**

The online version of this article (10.1007/s00216-018-1516-6) contains supplementary material, which is available to authorized users.

**Graphical abstract:**
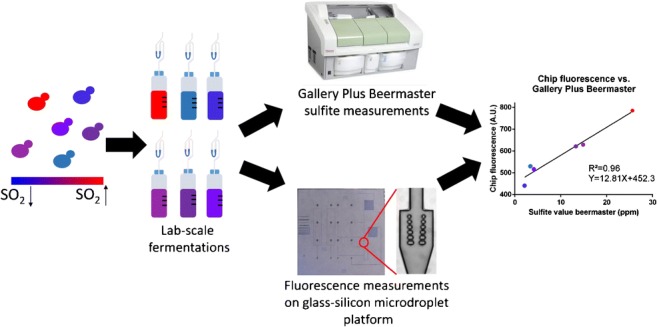
ᅟ

## Introduction

Yeasts are commonly used for the production of alcoholic beverages like beer, wine, and sake, where they produce alcohol, CO_2_, and thousands of secondary metabolites. One important byproduct of yeasts’ amino acid metabolism is sulfite, an antioxidant that delays flavor staling and inhibits the growth of various microorganisms, thereby prolonging the product’s shelf life. Exogenous sulfite is therefore sometimes added to these beverages and, specifically, to wine. However, concerns about the safety of sulfite have recently increased as it may cause respiratory problems or migraine in sulfite-sensitive patients [1]. For this reason, food products with sulfite levels exceeding 10 mg L^−1^ must be labeled with the statement “contains sulfites” [2]. A sensitive, accurate, and rapid analysis method to measure sulfite levels in fermented beverages is therefore desirable. While several methods to measure sulfites are available, including the Monier-Williams method, combining distillation and titration [3], gas chromatography [4], HPLC [3], ion exchange chromatography, amperometry [1], fluorescent probes [5], enzymatic methods [6], and colorimetry [7], many of these are time-consuming, require costly equipment, and the results can in some cases even be unreliable [2, 3]. One technique that can overcome the limitations posed by the currently applied methods is droplet microfluidics. Here, the sample is partitioned in picoliter droplets in a water-in-oil emulsion. Reagents (chemicals or enzymes) can be added by controlled merging of sample and reagent droplets [8–10]. This reduces the consumption of costly reagents and reaction time. Moreover, single cells can be encapsulated and cultivated in microdroplets to identify and isolate cells with specific desirable characteristics [11–13].

In this study, we discuss the development of a reusable glass-silicon microdroplet platform that allows measuring the sulfite concentration of beers within 3 min by integrating generation of surfactant-stabilized droplets, droplet destabilization by Pico Break 1, pillar-induced droplet merging for enzyme addition, emulsion restabilization, and fluorescence measurement with a photomultiplier tube. First, a fluorescence-based enzymatic assay was developed and characterized for its limit of detection (LOD), limit of quantification (LOQ), dynamic range and the influence of salt, food colorant, and sugars. Next, beers that were fermented with yeast strains that produce various amounts of sulfite were analyzed using the fluorescent assay in microplates and the standard colorimetric 5,5′-dithiobis-(2-nitrobenzoic acid) (DTNB) Gallery Plus Beermaster assay, indicating that both methods are in good agreement. The enzymatic assay was validated in microdroplets on the glass silicon chip, confirming that the platform can be used for sulfite analysis of fermentation samples. Although the presented platform can be used for the enzymatic analysis of microdroplets, stable reinjection of microdroplets that are fermented with a library of cells remains elusive as the fluidics’ entrances are equipped with a tight array of micropillars to prevent dust contamination. Therefore, removing the pillars in a future design could open up applications for high-throughput screening of microbes, plant, or mammalian cells. Moreover, the implementation of miniaturized pumps and optics would increase the automation and portability of the system, which would increase its user-friendliness.

## Materials and methods

### Reagents and enzymes

The following materials were purchased from Sigma-Aldrich (Diegem, Belgium): acetaldehyde (> 99.5%), citric acid monohydrate (> 98%), chloramphenicol (> 98%), d-glucose monohydrate (> 99%), d-maltose monohydrate (> 99%), and sodium metabisulfite (> 95%). Amplex Red, dimethyl sulfoxide (DMSO), horseradish peroxidase, and 5× sodium phosphate buffer (pH 7.4) from the hydrogen peroxide/peroxidase assay kit and SO_2_ total reagents for the Gallery Plus Beermaster were obtained from Thermo Fisher Scientific (Leuven, Belgium). Other products used in this study include 2% and 5% *w*/*w* 008-Fluorosurfactant in HFE7500 (Ran Biotechnologies, Beverly, USA), bacteriological peptone (Lab M, Bury, UK), (heptadecafluoro-1,1,2,2-tetrahydrodecyl)trimethoxysilane (95%) (abcr, Karlsruhe, Germany), malt extract light (7-12 EBC; Brouwland, Beverlo, Belgium), HFE7500 oil (3 M, Cergy, France), oenocyanin red wine extract (Vinoferm, Beverlo, Belgium), Pico Break 1 (Dolomite Microfluidics, Royston, UK), sodium chloride (VWR, Oud-Heverlee, Belgium), sucrose (Merck KGaA, Darmstadt, Germany), sulfite oxidase from the total sulfite assay kit (Megazyme, Wicklow, Ireland), and yeast extract (Lab M, Bury, UK).

### Lab scale fermentations

The sulfite production of four *Saccharomyces cerevisiae* (YV1-4), one *Saccharomyces eubayanus* (YV5), and seven *Saccharomyces pastorianus* strains (YV6-12) (Table 1) [14] was assessed in lab-scale fermentations. First, yeast was propagated by inoculation into 5-ml yeast extract-peptone-glucose *(*YPD; 1% *w*/*v* yeast extract, 2% *w*/*v* bacteriological peptone, and 2% *w*/*v*d-glucose monohydrate) medium at room temperature and 300 rpm. After 16 h of incubation, 1 ml of the culture was transferred to 50-ml yeast extract-peptone-maltose (YPM; 1% *w*/*v* yeast extract, 2% *w*/*v* bacteriological peptone, and 4% *w*/*v*d-maltose monohydrate) medium in a 250-ml Erlenmeyer flask and incubated at 20 °C and 200 rpm for 48 h. Next, 150 ml of 16°P beer medium (170 g/l malt extract light and 0.01% *w*/*v* chloramphenicol) was pitched with 10^7^ cells/ml. Beers were fermented with water locks on a stirring platform (150 rpm) for 10 days at 14 °C. After fermentation, samples were centrifuged and the supernatant was frozen for sulfite analysis.

**Table 1:** Yeast strains screened for sulfite production in lab-scale fermentations and their corresponding sulfite concentration as measured by the Gallery Plus Beermaster

Yeast number	Species	Sulfite production (ppm)
YV1	*S. cerevisiae*	25.60
YV2	*S. cerevisiae*	2.14
YV3	*S. cerevisiae*	2.45
YV4	*S. cerevisiae*	2.45
YV5	*S. eubayanus*	14.90
YV6	*S. pastorianus*	13.49
YV7	*S. pastorianus*	13.26
YV8	*S. pastorianus*	2.20
YV9	*S. pastorianus*	5.57
YV10	*S. pastorianus*	9.26
YV11	*S. pastorianus*	4.15
YV12	*S. pastorianus*	3.44

### Fluorescent measurement of sulfite in microplates

Thirty-five microliters of sample was transferred to a black microplate and 50 μl of reaction mix, containing 100 μM of Amplex Red (diluted from a 10 mM stock in DMSO), 0.2 U/ml horseradish peroxidase, and 0.5 μl sulfite oxidase dissolved in 0.05 M sodium phosphate buffer (pH 7.4), was added. The plate was sealed and fluorescence was measured after 2 min using the Tecan Infinite M200 Pro (560-nm excitation, 600-nm emission, bottom read, 25 flashes, and gain 78).

### Analytical performance of fluorescent sulfite measurements

The limit of detection (LOD) and limit of quantification (LOQ) of the fluorescent assay were measured by preparing calibration samples of sodium metabisulfite in 0.05 M sodium phosphate buffer (pH 7.4) ranging between 0 and 30 ppm. All samples were analyzed in triplicate, except the blank sample which had eight replicates. The fluorescent readout of samples was measured as described above. The LOD and LOQ were calculated as the average readout of the 0 ppm sample plus three or ten times the standard deviation on this average, respectively [15]. The sulfite concentration corresponding to the LOD and LOQ was retrieved from the regression curve that was made using the first seven data points. The linear dynamic range of the assay was determined by the LOD and the sulfite concentration at which a 5% deviation from a perfect linear curve could be detected. The dynamic range of the assay does not take linearity into account and was confined by the LOD and the sulfite concentration at which readouts reached a plateau [16].

### Influence of sugars, food colorant, and salt present in food products on the fluorescent readout

The influence of sugars, food colorant, and salt on the fluorescent readout was assessed by adding glucose (5 and 10% *w*/*v*), sucrose (5 and 10% *w*/*v*), oenocyanin red wine extract (0.1 and 0.5% *v*/*v*) and sodium chloride (0.5 and 1% *w*/*v*) to YV4 fermentation sample (Table 1). The samples were analyzed in triplicate using the fluorescence-based enzymatic assay. Readouts were normalized to the average of YV4 sample without added salt, colorant, or sugar, and the averages were compared to the untreated sample using unpaired *t* tests (*α* = 0.05).

### Sulfite measurement of fermentation samples with the Gallery Plus Beermaster

The sulfite concentration of the fermentation samples was measured using the SO_2_ total reagents in the Gallery Plus Beermaster. The Gallery Plus Beermaster was calibrated using standard solutions containing 0 to 50 ppm sodium metabisulfite, 0.5% *w*/*v* citric acid and 0.08% *v*/*v* acetaldehyde. 0.5 ml of sample was filtered using a 0.2-μm polyethersulfone syringe filter and transferred to the Gallery Plus Beermaster for analysis.

### Analysis of fluorescent sulfite measurements of fermentation samples

The sulfite-induced fluorescence of fermentation samples was measured in microplates using the earlier described assay. Fluorescence values were plotted against the corresponding Gallery Plus Beermaster values, and theoretical sulfite concentrations were calculated from the regression curve. A Bland-Altman comparison test [17] was performed to compare both analysis methods. Normality of the concentration differences measured between both methods was tested using a D’Agostino and Pearson normality test.

### Microfluidic chip fabrication

**Fig. 1:**
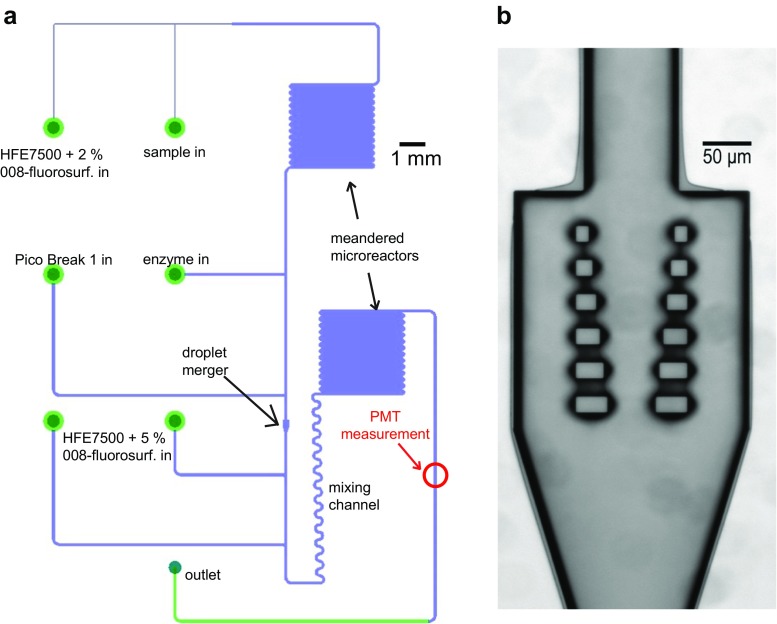
Glass-silicon microfluidic chip that allows generating and merging droplets. **A** Droplets containing a sample fluid (e.g., fermentation product) are generated in a T-junction and are alternated with enzyme-containing droplets. Next, the droplets are incubated in the first meandering microreactor, with the incubation times depending on the flow rate (which can be stopped altogether to allow extended incubation as needed). Next, the emulsion is destabilized by addition of Pico Break 1, after which droplets are merged in the merging cavity (see panel **B**). Next, the Pico Break 1 concentration is reduced by adding oil with 5% surfactant, allowing to restabilize the emulsion. After droplet mixing and 2 min of incubation in the second microreactor, droplet fluorescence is read using a photmultiplier tube (PMT). **B** Close-up of merging cavity. Sample droplets entering the merging cavity make contact with the pillars and stop flowing (see also [9]). Next, the consecutive enzyme droplet enters the merging cavity and merges with the immobilized sample droplet, after which the merged droplet leaves the merging cavity

The microfluidic chip (see Fig. 1a) was designed using Cadence Virtuoso. Fluidic inlets were lined with pillar filters to prevent channel clogging by dust particles. The fluidic channels are 100 μm wide, except at the droplet generator where dimensions are decreased to 30 μm and in the merging cavity where the channel dilates to 250 μm. This merging cavity contains six rows of two pillars that are removed 65 μm from the channel walls with a vertical spacing of 20 μm. The horizontal interpillar distance gradually decreases from 90 to 50 μm as the pillar size increases from 15 to 35 μm (see Fig. 1b) [9]. The fluidic pattern was transferred to a chrome-to-quartz mask and then to silicon using standard photolithography. Next, the channels were etched 30-μm deep in the silicon using deep reactive ion etching followed by the thermal growth of a 30-nm top layer of silicon oxide that easily withholds hydrophobic coatings, using the optimized process described by Majeed and coworkers [18]. Etching quality was evaluated using scanning electron microscopy, which indicated the channels were rectangular for a stable flow profile (see Electronic Supplementary Material (ESM) Fig. S1). Finally, the channels were sealed by anodic bonding with a 400-μm thick Pyrex slide. One hundred fifty-microliter deep inlet ports were made from the backside by a second deep reactive ion etching step. The microfluidic chip was made hydrophobic using (heptadecafluoro-1,1,2,2-tetrahydrodecyl)trimethoxysilane (FDTS) vapor coating by placing the chips together with 125 μl of FDTS for 2 h in a 125 °C vacuum oven. This was important to assure the stable formation of a water-in-oil emulsion as the silicon oxide top layer is hydrophilic and does not allow the stable formation of microdroplets without a hydrophobic coating [19] (see ESM Fig. S2 A-B). The coating quality was verified by spotting a 1-μl droplet on the chip’s surface before and after coating and by measuring the contact angle using the Contact Angle System OCA machine (Data Physics) and the SCA20U software (see ESM Fig. S2 C-D). Chips with contact angles exceeding 100° were considered of sufficient hydrophobicity.

### Sulfite measurement in microdroplets

Reagents for on-chip sulfite measurements were stored in glass Hamilton syringes that were mounted on syringe pumps (KD Scientific). Beer droplets (± 150 pl) were generated in a T-junction by creating an emulsion of beer sample (50 nl/min) in HFE7500 with 2% 008-fluorosurfactant (150 nl/min) that consistently avoids droplet merging [20]. Beer droplets were alternated with enzyme droplets in a second T-junction (90 nl/min; 50 μl of enzyme solution contained 100 μM Amplex Red (diluted from a 10 mM stock in DMSO), 0.2 U/ml horseradish peroxidase and 0.5 μl sulfite oxidase dissolved in 0.05 M sodium phosphate buffer (pH 7.4)). Next, the fluorosurfactant was destabilized by addition of Pico Break 1 (90 nl/min), allowing droplets of sample and enzyme to merge upon making contact in the merging cavity. The emulsion was restabilized by stepwise addition of fresh HFE7500 with 5% 008-fluorosurfactant (2 times 300 nl/min). The applied flow rates yielded droplets of good stability as described in [21]. The droplet content was mixed and incubated for 2 min in a meandered channel. The fluorescence readout was obtained using a photomultiplier tube (PMT) that was mounted on an Olympus IX-71 microscope supplied with a × 40 objective and Chroma 41002b wavelength filter. Peak integers of droplets were calculated using MATLAB. The integer values were plotted against the corresponding Gallery Plus Beermaster values, and theoretical sulfite concentrations were calculated from the regression curve. A Bland-Altman comparison test [17] was performed to compare the chip analysis with the Gallery Plus Beermaster method. Normality of the concentration differences measured between both methods was tested using a Kolmogorov-Smirnov test.

## Results and discussion

Lab-scale beer fermentations were performed to assess the sulfite production of 12 industrial yeast strains (4 *S. cerevisiae*, 1 *S. eubayanus*, and 7 *S. pastorianus* strains; see Table 1). After fermentation, the sulfite content of each bottle was measured using the Gallery Plus Beermaster total SO_2_ assay. This measurement relies on the reaction between sulfite and DTNB, yielding a molecule that absorbs light at 400–425 nm (ESM Fig. S3), and is considered a reliable method for sulfite analysis [22]. The test revealed that the different yeast strains produced variable amounts of sulfite, ranging from 2.14 to 25.6 ppm (Table 1).

### Fluorescent measurement of sulfite in microplates

Because absorbance or transmission-based measurements are difficult to perform on a non-transparent silicon device and are characterized by a low sensitivity, we developed an enzymatic assay yielding a fluorescent readout for SO_2_ measurements in droplets that is compatible with glass-silicon chips. This enzymatic assay consisted of (i) oxidation of sulfite into sulfate and hydrogen peroxide by sulfite oxidase and (ii) subsequent reduction of hydrogen peroxide and oxidation of Amplex Red into resorufin, a highly fluorescent compound, by horseradish peroxidase (ESM Fig. S4).

**Fig. 2:**
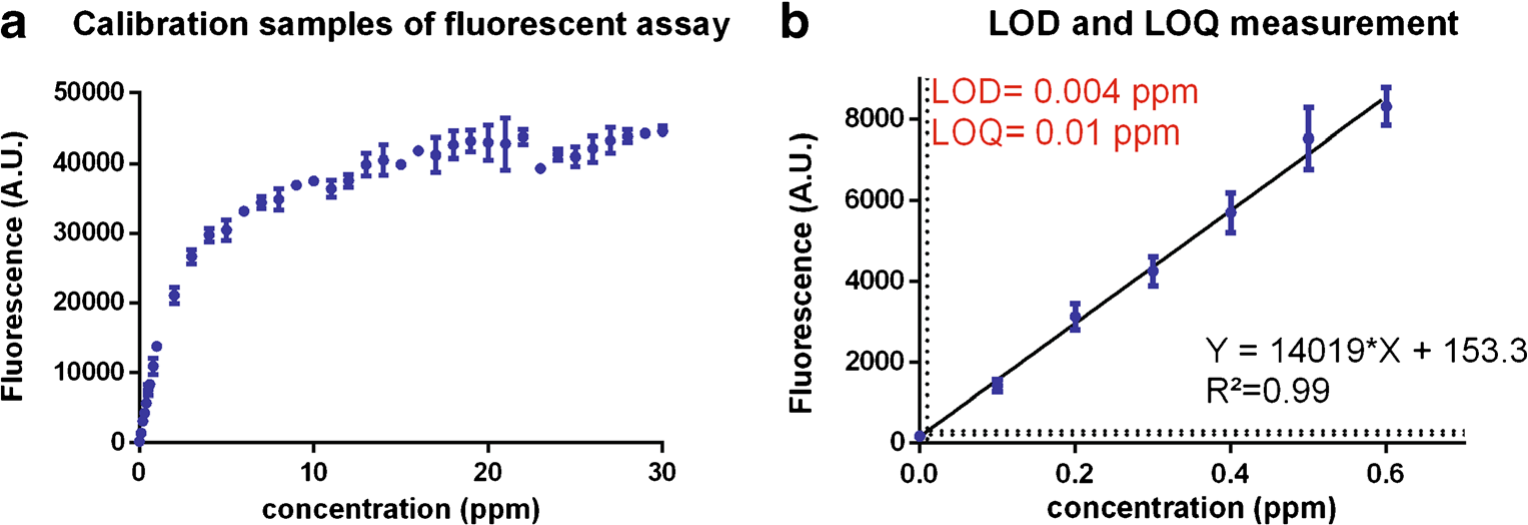
Calibration of the fluorescent assay and measurement of the LOD and LOQ. **A** Calibration samples containing 0 to 30 ppm were analyzed using the fluorescent assay. The calibration was perfectly linear between the LOD up to 2 ppm, which confined the dynamic linear range (see ESM Fig. S5). The dynamic range was not restricted by linearity and was confined by the LOD and the concentration at which the readout reached a plateau, which was around 10 ppm. **B** The LOD and LOQ were calculated as the readout for the 0 ppm sample + three or ten times its standard deviation. The corresponding sulfite concentrations were derived from the regression curve. As the LOD and LOQ were very low, it can be concluded that the fluorescent assay is very sensitive

A calibration was set up for this fluorescence-based assay by analyzing samples containing a range from 0 to 30 ppm of sodium metabisulfite (Fig. 2). The limit of detection (LOD) and limit of quantification (LOQ) of the assay were calculated as the average readout of the 0 ppm sample plus three and ten times its standard deviation, respectively. The sulfite concentrations corresponding to this LOD and LOQ were calculated from the regression equation plotted using the first seven data points and were 0.004 and 0.01 ppm for the LOD and LOQ, respectively (Fig. 2b). These low values indicated that the fluorescent assay was very sensitive. The dynamic linear range of the assay was confined by the LOD and the sulfite concentrations at which a 5% deviation from a perfect linear calibration could be detected, which was situated at 2 ppm (see Fig. 2a and ESM Fig. S5). As fluorescence measurements are expected to have a dynamic linear range of at least two orders of magnitude, the assay is reliable for sulfite measurements [16, 23]. In contrast to the dynamic linear range, the dynamic range is not restricted by linearity and is limited by the concentration at which readouts reach a plateau, which was situated around 10 ppm.

**Fig. 3:**
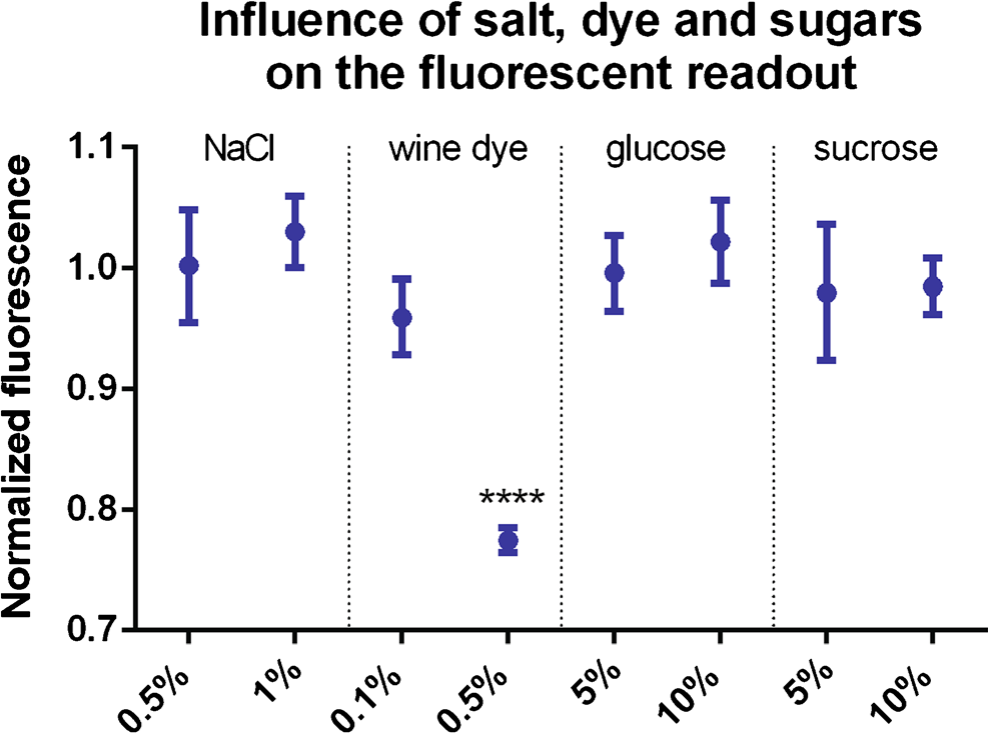
Influence of salt, food colorant, and sugars on the fluorescent readout. Sodium chloride (0.5 and 1% *w*/*v*), red wine colorant (0.1 and 0.5% *v*/*v*), glucose (5 and 10% *w*/*v*), and sucrose (5 and 10% *w*/*v*) were added to the YV4 fermentation sample containing 2.45 ppm of sulfite. The samples were analyzed using the fluorescence-based enzymatic assay and their readout was normalized to the sample without any addition. Sodium chloride, glucose, and sucrose did not influence the readout. Addition of red wine colorant significantly lowered the fluorescence measured as part of the photons that excited Amplex Red could be absorbed by the anthocyanins present in the red wine colorant, thereby lowering the amount of emitted photons [24]

A second performance element of the fluorescent assay that was assessed is the possible influence of salt, colorants, and sugars that could be present in beverages on the readout. This was investigated by adding different concentrations of sodium chloride, red wine colorant, glucose, and sucrose to the YV4 fermentation sample, which had a sulfite concentration in the dynamic range of the assay (2.45 ppm; Table 1). The readouts were compared to the sample without additions, indicating that sodium chloride, glucose, and sucrose did not inhibit the assay (Fig. 3). Red wine colorant significantly lowered the readout at a concentration of 0.5% *v*/*v* as the anthocyanins present in this colorant absorb the 560-nm light used to excite Amplex Red, thereby lowering the amounts of photons that could be emitted and detected [24]. This means that calibration samples containing red wine colorant need to be ran before red wine samples can be analyzed with the assay.

**Fig. 4:**
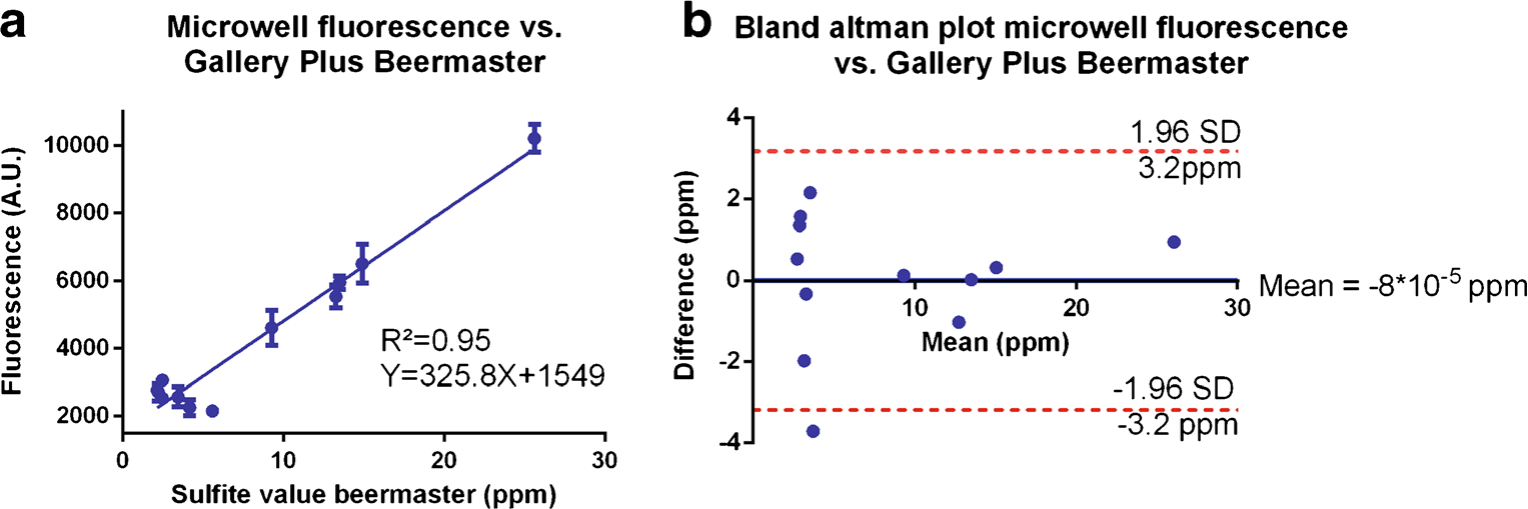
Fluorescent sulfite measurement in microplates. **A** Correlation of fluorescence values to sulfite concentrations measured using the standard Gallery Plus Beermaster assay. Error bars depict the standard error of the mean (*n* = 3). Fluorescence and Gallery Plus Beermaster values are strongly correlated. **B** Bland-Altman comparison test of both analysis methods confirms that the two assays yield similar results. Theoretical sulfite concentrations were calculated via the regression curve. The difference in sulfite concentration measured between both methods was plotted against the mean concentration. The differences in sulfite concentration were normally distributed according to a D’Agostino and Pearson normality test (*P* = 0.1247). Red dashed lines depict the acceptable level of error (mean difference ± 1.96 × standard deviation of differences). Both analysis methods showed good accordance for 11 out of 12 samples, with one sampling just falling outside of the target confidence interval

After the analytical performance of the assay was measured, all fermentation samples were analyzed with the fluorescent assay in microplates and the readouts were correlated to the absorbance-based sulfite measurements using the Gallery Plus Beermaster (Fig. 4). Both measurements yielded very similar results (*R*^2^ = 0.95). A Bland-Altman comparison test [17] was performed to further confirm the agreement between the two assays. Therefore, theoretical sulfite concentrations for the fluorescent assay were calculated using the regression equation. The concentration difference measured between predicted values from the regression curve and Gallery Plus Beermaster values was plotted against the mean for these concentrations. Differences measured between both methods were normally distributed according to a D’Agostino and Pearson normality test (*P* = 0.1247). A 95% confidence interval for the concentration differences was calculated, and 11 out of 12 fell within the interval. On average, the relative difference between measurements obtained by the two methods declines as sulfite concentrations increased and fell below 1 ppm for samples with sulfite concentrations higher than 10 ppm, the lowest concentration at which labeling is obliged.

### Sulfite measurement in microdroplets

**Fig. 5:**
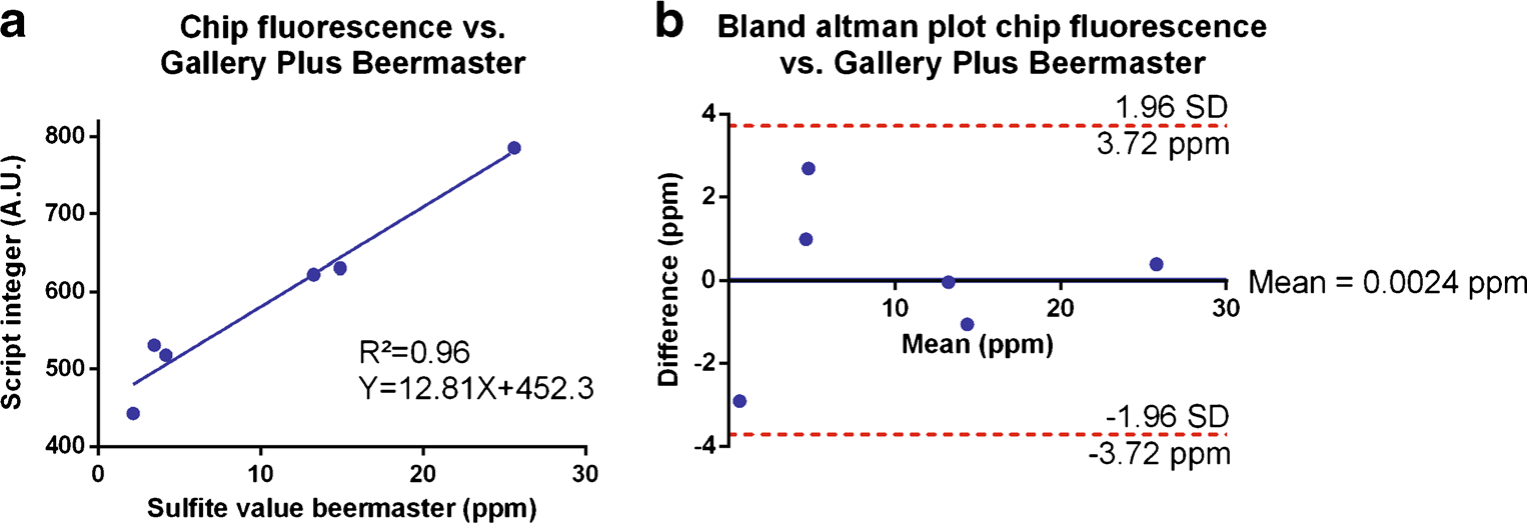
Sulfite measurement on the microdroplet platform. **A** Correlation of the PMT peak integers to sulfite concentrations measured by the DTNB method on the Gallery Plus Beermaster. Error bars depict the standard error of the mean (*n* = 300) and vary between 3 and 7 arbitrary units. Microdroplet and Gallery Plus Beermaster measurements are strongly correlated. **B** Bland-Altman comparison test of the chip and the DTNB assay shows that both assays yield similar results. Theoretical sulfite concentrations were calculated via the regression curve. The difference in sulfite concentration measured between both methods was plotted against the mean measured concentration. The differences in sulfite concentration were normally distributed according to a Kolmogorov-Smirnov test (*P* = 0.20). Red dashed lines depict the acceptable level of error (mean difference ± 1.96 × standard deviation of differences). All points were situated within this confidence interval. Therefore, the microdroplet platform cannot be discriminated from the Gallery Plus Beermaster for sulfite measurements

After we confirmed that the fluorescent assay yielded very similar results as the standard absorbance assay using the Gallery Plus Beermaster, we transferred the fluorescence-based assay to a microdroplet platform and analyzed six samples with variable sulfite concentration (YV1-2, YV 5, YV7, and YV 11-12; Table 1). Surfactant-stabilized droplets containing the sample solutions were generated in a T-junction, and droplets containing the enzyme solutions were interspersed in-between the sample-containing droplets. Next, the emulsion was destabilized by addition of Pico Break 1 to facilitate merging of one sample- and one enzyme-containing droplet in a pillar-merger element similar to the one described by Niu and coworkers [9]. After droplet merging, the emulsion was stabilized again by addition of oil and 5% surfactant and fluorescence of 300 droplets was measured using a PMT. The PMT peak integers correlated to the sulfite concentrations measured by the Gallery Plus Beermaster (Fig. 5a; *R*^2^ = 0.96). Both analysis methods were compared with a Bland-Altman comparison test as was done for the microplate analysis [17]. Theoretical sulfite concentrations were calculated from the regression curve (Fig. 5a), and the concentration differences measured between both methods were plotted against the mean concentration measured for both methods. A Kolmogorov-Smirnov test showed normal distribution of the differences measured between the chip and the Gallery Plus Beermaster assay (*P* = 0.20). All measurements obtained by the two different methods fell within a 95% confidence interval (Fig. 5b), indicating that both analysis methods yield the same results. As microdroplets have been extensively used for high-throughput phenotyping of bacterial and fungal cells [11–13], it would be valuable to use the microdroplet platform for reinjection of droplets that are cultivated with a library of microbes. However, as the fluidics’ entrances are equipped with a tight array of micropillars to prevent channel clogging by dust particles (see ESM Fig. S6), droplets cannot be stably reinjected in the presented platform. Removing the pillars in a future version and integrating a droplet sorting mechanism that allows to isolate droplets containing the cells of interest would therefore open the door for many high-throughput droplet-based single-cell screenings. Other optimizations that could be implemented in the presented platform and would facilitate its use by increased automation and portability are the implementation of miniaturized optics and pumps [25, 26].

## Conclusion

We developed a reusable microdroplet platform that allows to measure sulfite concentrations in extremely small samples of fermentation products and only requires 3 min of analysis time per sample. In brief, the platform allows the generation of sample droplets interspersed with droplets containing an enzyme solution. Next, the emulsion is destabilized by addition of Pico Break 1 and droplet merging is induced by micropillars as described by Niu and coworkers [9]. After restabilization of the emulsion by addition of oil and surfactant, droplets are incubated for 2 min to allow the enzymatic assay to complete, and the fluorescence of each droplet is subsequently measured using a PMT. Our analyses show that the fluorescent assay was very sensitive with a LOD of 0.004 ppm and that the assay had a dynamic range of three orders of magnitude. While addition of salt, glucose, or sucrose did not affect the assay, colorants could influence the readout, especially at low sulfite concentrations, likely because anthocyanins can absorb part of the photons that are used to excite the fluorescent target molecules. Therefore, colored beverages like red wine need specific calibration curves for their analysis. Droplet-based measurements yielded the same results as the established macroscale analysis method using the DTNB method of the Gallery Plus Beermaster. Droplet reinjection for cell-based droplet screenings currently remains elusive as the fluidics’ entrances are equipped with a tight array of pillars that prevents stable reinjection of droplets. Therefore, the integration of a droplet sorter mechanism, miniaturized optics, and pumps would open the door for high-throughput screenings of cell libraries in an automated and portable platform.

## Electronic supplementary material

ESM 1 (PDF 819 kb)
