# Supplementary material for: Development and validation of a glass-silicon microdroplet-based system to measure sulfite concentrations in beverages
Source: Anal Bioanal Chem. 2019 Jan 14;411(6):1127–34. doi: 10.1007/s00216-018-1516-6 (PMC6373184; doi:10.1007/s00216-018-1516-6)
Supplement: Supplementary file 1 — (PDF 819 kb) [file 216_2018_1516_MOESM1_ESM.pdf]

Analytical and Bioanalytical Chemistry

Electronic Supplementary Material

Development and validation of a glass-silicon microdroplet-based system to measure sulfite concentrations in beverages

Yannick Vervoort, Rodrigo Sergio Wiederkehr, Michiel Smets, Maarten Fauvart,
Tim Stakenborg, Gabrielle Woronoff, Liesbet Lagae, Kevin J. Verstrepen

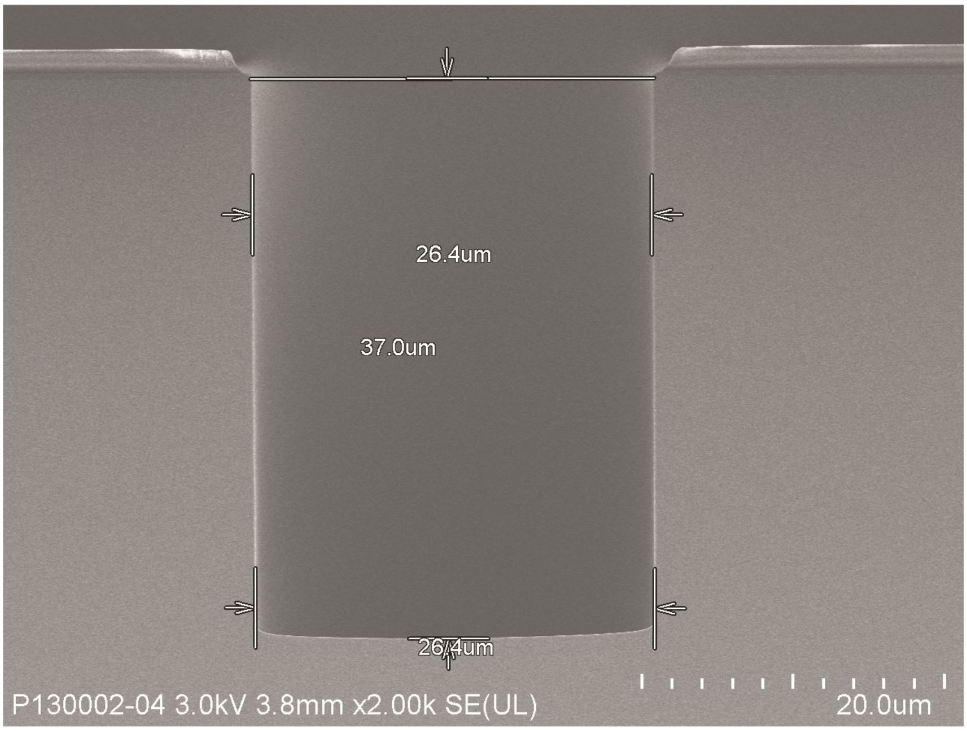

Fig. S1 Scanning electron microscopy picture of the droplet generator on the silicon chip after deep reactive ion etching. The channels of the batch shown were etched 37 µm deep and 26.4 µm wide. Channels were rectangular, giving optimal stability for the flow profile

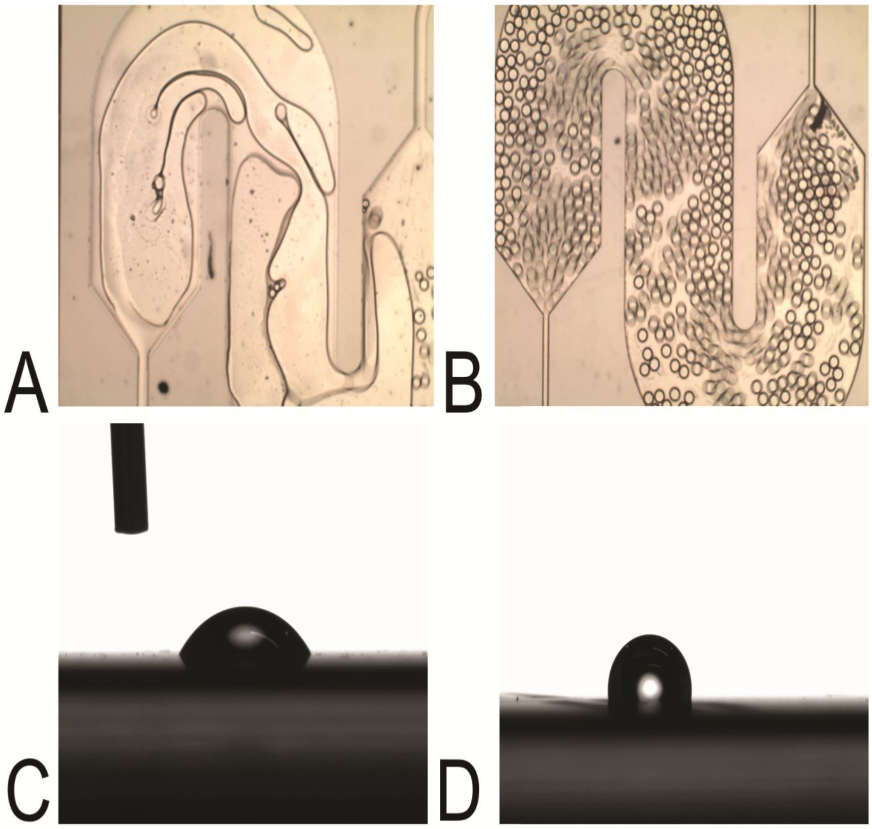

Fig. S2 Hydrophobic coating of glass-silicon chips. **A.** Chips without FDTS coating do not allow to generate a water-in-oil emulsion. **B.** Droplet formation after FDTS coating. **C-D.** Contact angles between the chip's surface and a 1µl water droplet were measured before (C; 60°) and after (D; 109°) FDTS coating to evaluate the coating quality. Chips with contact angles exceeding 100° were considered of good quality

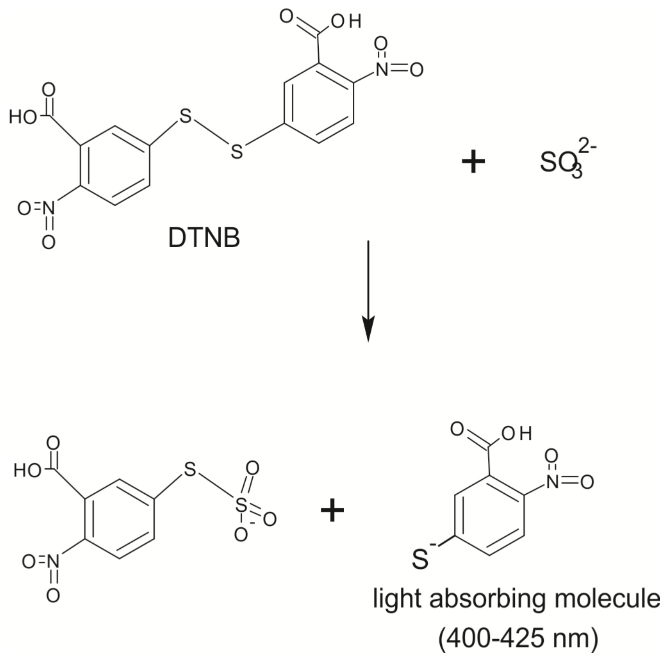

Fig. S3 Absorbance-based detection of sulfite. Sulfite can be measured by chemical reaction of 5,5'-dithiobis-(2-nitrobenzoic acid) (DTNB) with sulfite, which generates a molecule that absorbs light between 400-425 nm

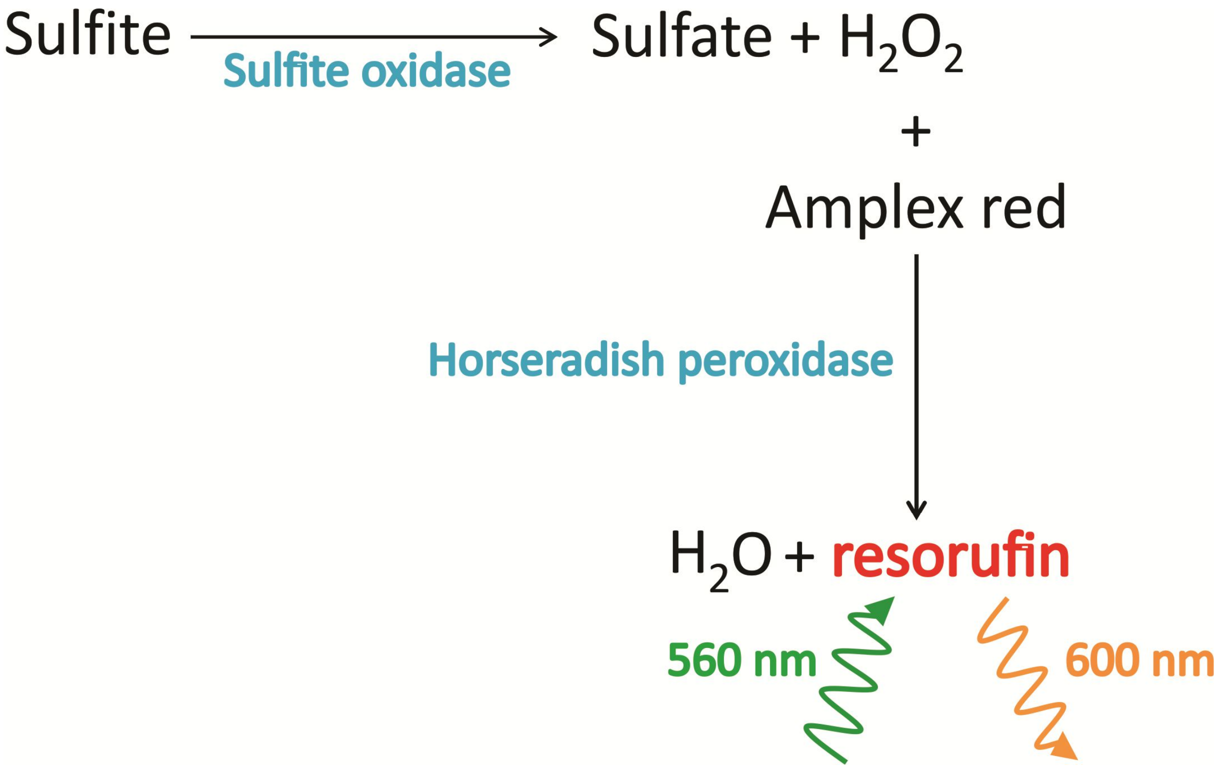

Fig. S4 Enzymatic assay for fluorescent sulfite measurement. Sulfite oxidase converts sulfite into sulfate, producing hydrogen peroxide. Horseradish peroxidase reduces this hydrogen peroxide into water while oxidizing amplex red into resorufin, a highly fluorescent compound

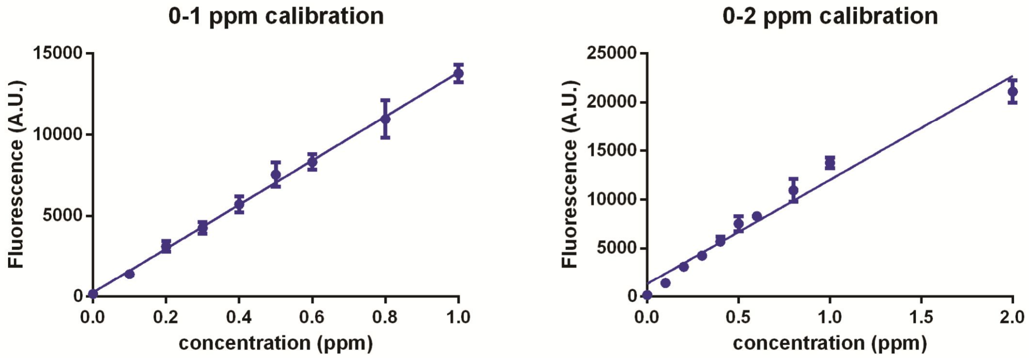

Fig

Fig. S5 Dynamic linear range of the fluorescence-based enzymatic assay. Linear plots were constructed between calibration samples of 0-1 and 0-2 ppm. In the latter, samples were situated on a nearly perfect linear curve. The 2 ppm sample deviated from this perfect linear curve, indicating that 2 ppm limits the dynamic linear range

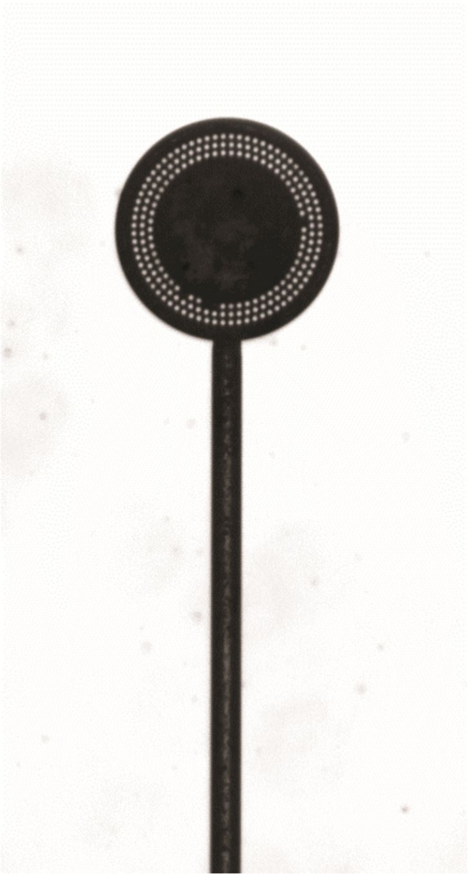

Fig. S6 The fluidics' entrances of the platform are equipped with micropillars to prevent dust contamination and clogging of the channels. The micropillars do not allow to stably reinject an emulsion containing microdroplets fermented with a library of cells. Therefore, a future design without the micropillars could be used for high-throughput screening of microbes, plant or mammalian cells
